# Investigation into the Impact of Online Learning and the Pandemic on Student Use of Mechanistic Arrows

**DOI:** 10.1021/acs.jchemed.4c01274

**Published:** 2025-04-08

**Authors:** Veeda Scammahorn, Samantha Houchlei, Hunter Williams, Melanie M Cooper

**Affiliations:** † Department of Chemistry, Michigan State University, 578 South Shaw Lane, East Lansing, Michigan 48824, United States; ‡ Department of Chemistry, University of Minnesota Twin Cities, Minneapolis, Minnesota 55455, United States

**Keywords:** High School/First Year/Second Year Undergraduate, Chemistry Education Research, Organic Chemistry, Reaction Mechanisms

## Abstract

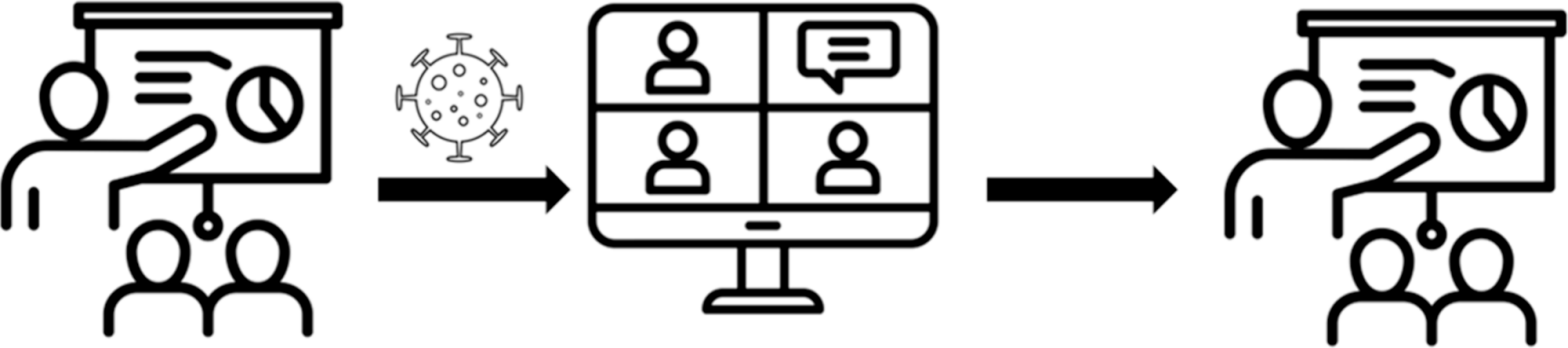

The impact of the COVID pandemic on student learning is still being felt more than two years after most classes returned to face-to-face instruction. In this study we investigate how the pandemic and the subsequent return to in-person instruction in an organic chemistry course impacted student performance on a pair of tasks for which we have historical data from pre-COVID courses. These tasks require students to draw mechanisms and predict products for two reactions: (1) a familiar reaction that students have been explicitly taught and (2) a reaction that requires students to use their knowledge to predict how an unfamiliar starting material will behave Analysis of the student responses for the familiar task showed that the 2022 (COVID cohort did not perform as well as in earlier studies), but by spring 2023 post COVID students had returned to a more normal pattern of performance that aligned well with our historical pre-COVID data (2018). In contrast, for the historically more difficult unfamiliar reaction, there was no significant difference among the cohorts’ ability to draw a plausible mechanism and predict a product over the three years of the study. That is there appeared to be a cadre of students who were able to complete this task despite the stress of a pandemic and changing instructional modalities. However, the percentage of students who were able to complete this unfamiliar task is typically less than 50% of the total. The implications of these findings are discussed.

## Introduction

### Overview

In March of 2020, as a result of the COVID-19 pandemic, most educational institutions in the United States made a rapid transition to online learning in its many forms. This necessity spurred numerous changes in instructional practices, some of which were retained when in-person learning returned.^1−3^ Now over three years later and with a return to “normal” there has been a great deal of concern about the impact of the pandemic on students, including what they have learned and retained, as well as their motivation and mental health. At the time of writing, studies seem to show that many students’ academic achievement was adversely impacted by the pandemic, particularly in math and STEM.^1^ While estimates of “learning losses” vary by country, and affected population,^4,5^ there is general agreement that over four years out from the start of the pandemic, many students had not yet caught up with their prepandemic peers, to say nothing of the lasting impact on student well-being.

In this study, in order to investigate the impact of the COVID pandemic on one aspect of student learning, we make use of data collected from an ongoing longitudinal study to compare college student performance on a set of organic chemistry activities that involve drawing mechanisms for both known and unknown organic reactions. We were able to collect data pre, during, and post COVID pandemic, and here we present our findings for two such reactions.

## Background and Prior Work

### Research on Impact of the Pandemic on Student Performance

As noted above, there appears to be general agreement that the impact of the pandemic and concurrent changes in instructional modes has led to a decline in many students’ learning, a widening of the achievement gap between rich and poor communities, and fears that for many children the impact of COVID may be a potentially permanent setback.^6^ A meta-analysis conducted in 2023, concluded that student achievement in math and science was particularly affected, and that one year after the pandemic restrictions were lifted many students had still not recovered from their learning loss.^1^ The impact of the pandemic was further exacerbated by inequities such as socioeconomic class, and resource availability.^2^ These large-scale studies on student learning are focused on precollege learners. In higher education, while there have been numerous publications dedicated to the changes to instruction made during the rapid transition to online learning (with over 600 papers published in the Journal alone), and studies on student perceptions of learning during the online courses,^3^ there are few (if any) studies in chemistry that report on learning outcomes over the period of the pandemic and subsequent return to in-person instruction.

### Prior studies on mechanism drawing

One of the most important activities that students encounter in organic chemistry courses is the construction of organic reaction mechanisms,^7^ which can be considered as the scientific practice of constructing a model that is both predictive and explanatory.^8^ Such models also require that students engage in mechanistic reasoning in a broader sense. As Russ and co-workers discuss “...mechanisms account for observations by showing that underlying objects cause local changes in the system by acting on one another”.^9^ Indeed in 2013 a national survey of organic instructors found that the purpose of mechanistic reasoning using the electron pushing formalism was “to explain and predict outcomes of chemical processes”.^7^ Consequently, we might expect students to use mechanistic arrows to both predict the product of a reaction and explain how that product is formed as the electrons rearrange. That being said, the organic chemistry education literature is replete with studies that show students have a great deal of difficulty with these tasks.^7,10−14^ Dood and Watts^15^ published a scoping review of this literature, and found that it could be parsed into four main challenges: (1) a product oriented focus rather than a process oriented approach, (2) difficulty in using the implicit information encoded in the molecular structures and electron pushing formalism, (3) difficulty in using multiple representations and (4) difficulty in reasoning with multiple variables. Bhattacharrya and Bodner showed that even graduate students tended to “push” electrons that “get me to the product”, without considering whether the arrows made mechanistic sense.^16^ Flynn has expanded this work to investigate how the different affordances of the electron pushing formalism (EPF) impact student performance.^12,13^ This study showed that students are better able add electrons when given the product, than are able to predict the product using the EPF.

It is certainly not surprising that there are so many studies on how students use the EPF and, and therefore it is not surprising that these studies have uncovered numerous problems. A great deal of implicit information is encoded in the drawing of an arrow from electron source to sink. For example, students must be able to predict how the structure of the reactants^17^ (and products) determines the electron distributions and therefore their properties. Ideally using the EPF is a natural consequence of decoding this implicit information such that predicting a mechanism involves not only a description of what is happening, but also why it is happening.^18^

In our work we have attempted to construct approaches that support student understanding of the underlying principles to emphasize the relationship between drawing mechanistic arrows, constructing models, and mechanistic explanations. To do this chemistry education researchers have emphasized mechanistic explanations for phenomena,^19−21^ and explicitly linked them to constructing explanatory and predictive models for organic chemistry reactions.^18^ We have used this relationship extensively in our curriculum development work, especially in the development of a transformed organic chemistry course “Organic Chemistry, Life, the Universe and Everything” (OCLUE).^22^

### Prior Studies upon Which This Report Is Based

This study builds on several of our prior studies on how students draw mechanisms of organic reactions. The first studies conducted in 2010^23,24^ reported on how students use of mechanistic arrows changes over the course of two semesters, and whether mechanism drawing is correlated with predicting an appropriate product. We found that for known reactions, use of mechanisms did not appear to correlate with predicting the product; however, for a reaction that students had not seen before, those who used mechanism drawing as a predictive tool were more likely to predict a chemically reasonable product. A crucial finding, in these early studies, was that less than 10% of students were able to make a reasonable prediction about the product of the unfamiliar reaction.

These studies were followed up almost ten years later in a pair of papers where we compared matched cohorts of students enrolled in two different curricula:^18,25^ A Traditional curriculum that followed a commercial text and was essentially the same as the curriculum used in the earlier study, and the Transformed curriculum, OCLUE. In these studies, we found that (1) there was no difference between the two cohorts in their ability to predict the outcome of a known reaction, (2) OCLUE students were more likely to draw mechanisms for all reactions, and (3) OCLUE students were more likely to predict the outcome of an unknown reaction. These studies taken together, conducted over a decade apart, at two different universities, showed remarkable consistency for students enrolled in traditional courses. However, students in the Transformed OCLUE course showed strikingly different patterns of behavior to those in Traditional courses, in that they drew mechanisms more consistently and were far more likely to use those mechanisms to predict the outcome of an unknown reaction.

### Research Questions

All of this previously published data provided us with the ability to investigate the use of mechanisms to predict products for students who learned organic chemistry during, and after the COVID pandemic compared to students who had taken organic chemistry prior to the pandemic. We used these data to answer two research questions that focus on comparing student performance pre, during, and postpandemic:

1. How do the responses for the familiar reaction vary over the three years of the study (2018 (prepandemic), 2022 (during-pandemic), and 2023 (post- pandemic))?
2. How do the responses for the unfamiliar reaction vary over the three years of the study (2018 (prepandemic), 2022 (during-pandemic), and 2023 (post- pandemic))?

In this paper we use the previously published data from spring 2018^18^ for OCLUE students and compare it to OCLUE student performance in spring 2022, when students had had two full years of online chemistry instruction, and spring 2023 when students who had returned to face-to-face instruction. In contrast to earlier studies, we do not include traditional students in this analysis for two reasons: 1) we already know that those students are typically unable to predict a mechanism and product for the unfamiliar reaction, and 2) problems with scheduling and access were more difficult during the pandemic.

## Methods

### Student Participants

This study was conducted in a large midwestern research intensive public university. All students in this study were enrolled in two semesters of a transformed course, OCLUE which has been described in earlier publications,^18,22,25,26^ and typically enrolls a majority of students who are in a preprofessional health or biology programs. In this study we compare student data from three years (spring 2018, spring 2022 and spring 2023), when the second semester of the transformed OCLUE course was taught. The study was determined as exempt (IRB# x13–787e Category: Exempt) by the Institutional Review Board of Michigan State University; students were informed of their rights and provided consent to use their anonymous data in this study.

The same instructor taught the students whose data were used for this new study during all three years for both semesters. During this time students were enrolled either in the OCLUE curriculum or in sections of the course that used a commercial textbook and mostly summative assessment strategies which we have previously labeled Traditional. There was some “crossover” of students between Traditional and OCLUE curricula after the first semester (that is, some students were enrolled in the Traditional section for OChem 1 in the fall and then enrolled in OCLUE for OChem 2 in the spring) as discussed in a prior study.^25^ However, for the purposes of comparisons among pre-, during-, and postpandemic cohorts in this study, we include only students who had each completed two semesters of OCLUE. Additionally, and in accordance with Houchlei^18^ we removed a small number of students who did not engage with the prompts (2 students in 2022 and 3 students in 2023). We also removed students who drew a product before drawing their mechanistic arrows (4 students in 2022 and 1 student in 2023). This procedure resulted in the responses from 165 students from 2018, 60 from 2022, and 96 from 2023 being incorporated into the study.

To ensure that the three groups of students were similar we compared several academic and demographic measures using a Mann–Whitney U test and calculated the effect size for any differences using Cramer’s V. We did not find any differences among the cohorts for ACT scores, or in self-reported binary gender distributions. There were small differences among average and course GPA, but we attribute this to the ways that grades were recorded during this time. For example, students were allowed to choose whether to accept their grade or to have it recorded as “pass”, drop dates were extended throughout the semester, and through spring 2023 students were allowed to opt for a credit/no credit record of their grade. Therefore, overall grade comparisons between pre, during and post-COVID would not provide productive information about students’ performance, or equivalence. Supporting Information S1–S5 provide a summary of all statistical analyses, which were performed in SPSS.^27^

This course is taught in large enrollment sections of 360, with three 50 min lecture periods a week, and one recitation session taught by graduate student assistants. The same text, learning objectives and course outline were followed as previous years; however, the shift to online learning during the COVID pandemic which affected years 2020–2022 necessitated some changes in course delivery and activities as outlined below and shown in Table 1.

**Table 1:** Instructional and Assessment Strategies for OCLUE 2018, 2022, and 2023 and Percent Contribution to Course Grade

Strategy	Instruction	Summative Assessments	Recitation	Homework	Attendance	Final Project
2018	In-person	In-person 65% 3 Midterms and final	In-person 15% Group work, 1/2 credit for attendance and 1/2 credit for effort	Online 15% Credit for good faith effort	In-person 5% Clickers	N/A
2022	Remote Synchronous and asynchronous	Online 51% Best of 4 midterm exams	Online 20% Synchronous	Online 20% Credit for good faith effort	N/A	Online 9% Based on effort
2023	Hybrid, in- person, remote Synchronous and asynchronous	Online 51% Best of 4 midterm exams	In-person and remote 20%	Online 20% Credit for good faith effort	N/A	Online 9% Based on effort

The pre-COVID 2018 course was taught in person, with 65% of the grade coming from summative assessments, and 35% from formative assessments on homework and recitation and class attendance points. After the switch to online, the class was taught on Zoom (both synchronously and asynchronously – class attendance was not required). 51% of the final grade was determined from summative assessments and the rest of the grade from formative tasks (homework and recitation), and a final “project” in which students were given a particular compound and asked questions that were intended to connect course material to a new situation. In 2023 some of the innovations were retained, but the main class activities moved back to in-person instruction. We should note that the spring of 2023 semester was interrupted by campus violence,^28^ which meant that a number of students elected to return to online only instruction. For all online activities except the time constrained summative assessments, students were allowed to use course resources. For the final online projects students were asked to cite references for material that they used in their responses.

### Prompt Timing and Administration

**Figure 1:**
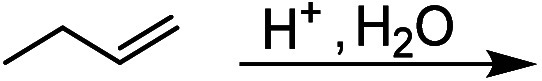
Familiar reaction prompt as it appears to students on beSocratic.

**Figure 2:**
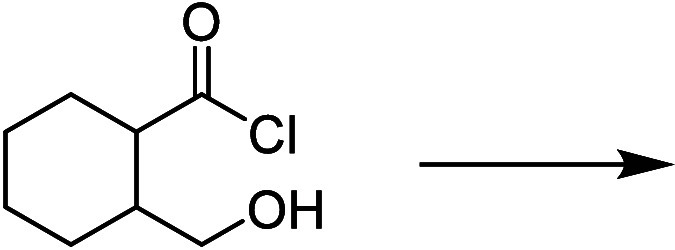
Unfamiliar reaction prompt as it appears to students on beSocratic.

As previously reported, the prompts were administered using beSocratic,^29,30^ the online formative assessment system that allows us to capture and record student drawing and writing. The familiar prompt, shown in Figure 1, was administered twice, both at the beginning and the end of the spring semester, while the unfamiliar prompt, shown in Figure 2, was administered at the end of the spring semester. In spring 2018 and 2022 all prompts were administered as extra credit assignments, as was the first administration in 2023, whereas the last administration in 2023 was part of the final project. At all time points all students were rewarded credit based on their good faith effort. We will discuss the implications of this in the results and discussion sections.

### Data Analysis

Student responses were deidentified and then replayed on beSocratic. We used the previously published, expanded coding scheme from Houchlei et al. that was designed to capture the various methods students used to draw mechanisms and predict a plausible product for the familiar and unfamiliar reaction. The coding schemes for these reactions can be found in the Supporting Information S8–S11. For each step of the mechanism each arrow was coded as plausible or not plausible. Plausible meaning that the arrow started from an electron source and ended at an electron sink that made mechanistic sense for the reaction. If the student drew all arrows incorrectly, they received the “Incorrect or No arrows drawn code”. Products were coded as plausible (either major or minor products were accepted) or not plausible.

**Figure 3:**
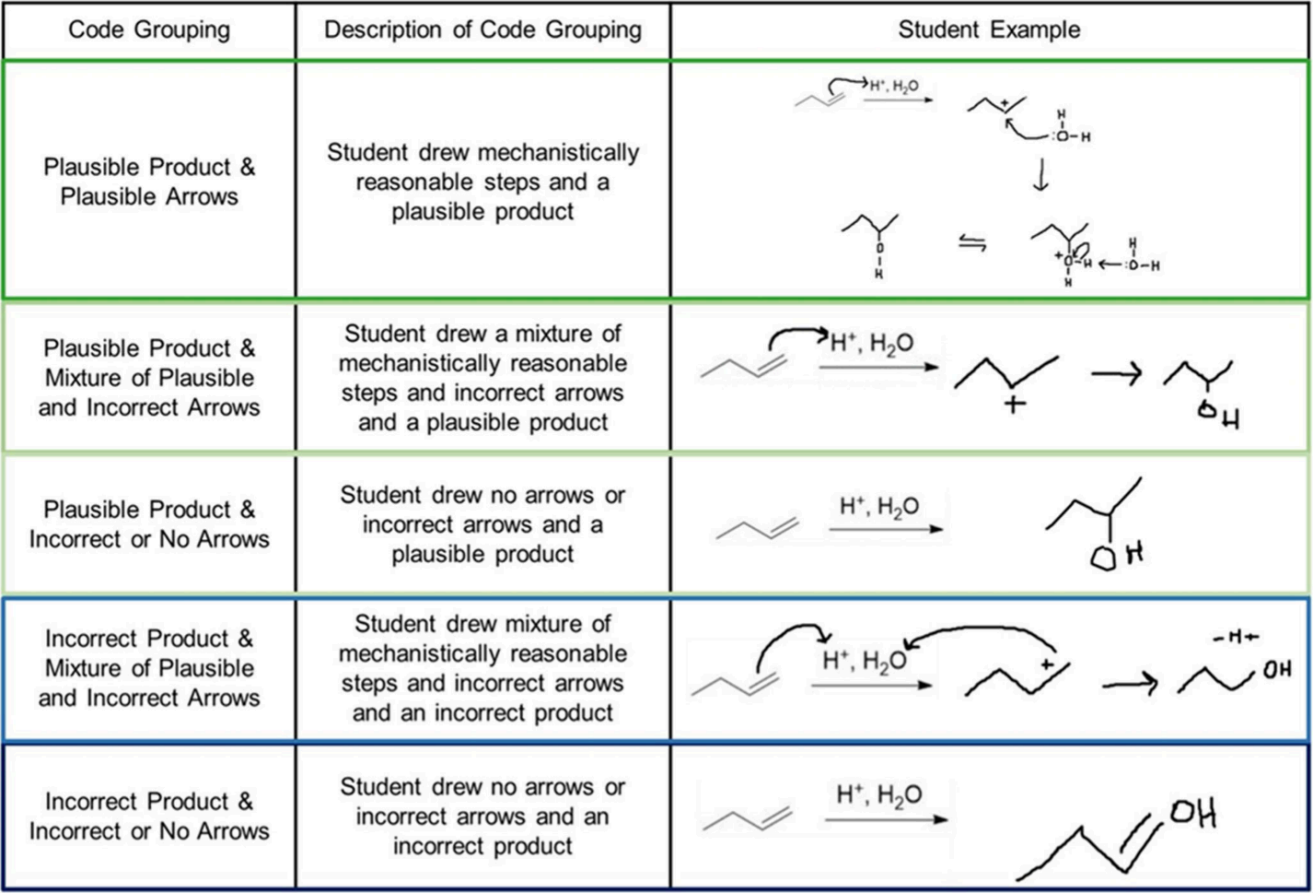
Groups assigned after coding of arrows and products. Each group is represented by an associated color. The green colored based boxes represent the set of students who drew a plausible product from the reaction, and the variations represent how correct their responses were. For example, dark green represents the group of students who drew a plausible product using a complete plausible mechanism, whereas the next lighter shade of green codes includes students who had a plausible product and some plausible arrows (and either incorrect or missing arrows). The blue colored based boxes represent the set of students who did not draw a plausible product for the reaction. Reproduced from ref (18). Copyright 2021, American Chemical Society.

Depending on the reaction, the number of possible mechanistic steps ranged from 7 to 22 for the familiar and unfamiliar reactions, respectively. An example of a pathway recorded in beSocratic is shown in Figure 3. Additionally, a small number of students (less than 10) did not answer the prompt or drew random lines. These students were removed from the data set.

Plausible reaction schemes for the familiar and unfamiliar reactions are shown in the Supporting Information (S6, familiar and S7, unfamiliar). To determine Inter-rater reliability (IRR) for each code two researchers (author) V.S. and undergraduate student, H.W., separately coded 20 responses, compared codes, and came to a consensus if any differences were found. Both coders then coded 50 new responses and compared to obtain kappa values. Cohen’s Kappa values ranged between 0.75 and 1.0 for both reactions, any disagreements were then discussed until agreement was achieved. The IRR data is shown in the Supporting Information S8–S11.

These analytical codes then allowed us to assign the students to one of five groups as described by Houchlei^18^ and shown in Figure 3.

### Statistical Analysis

Chi square tests were used to examine the relationship between students in their respective cohorts and those assigned to groups shown in Figure 3, The Chi square test compares the observed frequencies of responses with the expected frequencies under the null hypothesis that the two variables are independent. This test was chosen to determine any differences between cohorts (2018, 2022, and 2023). Results for these tests can be seen in Tables 2–7. Additionally, to avoid a Type 1 error, a Bonferroni correct was applied to account for the 18 pairwise comparisons made between the cohorts (2018, 2022, and 2023) adjusting the significance level to *p* < 0.002 (0.05/18 comparisons).

**Table 2:** Time Point 1 Familiar Reaction: Comparison of the Percent of Students Who Drew a Plausible Product for 2018, 2022, and 2023^a^

Cohort (count/total n)	Cohort (count/total n)	χ2 (df = 1)	p-value	Cramer’s V
2018, 59% (97/165)	2022, 27% (16/60)	18.16	<0.001*	0.284 (small – medium)
2018, 59% (97/165)	2023, 42% (40/96)	7.134	0.008*^+^	0.165 (small)
2022, 27% (16/60)	2023, 42% (40/96)	3.610	0.057*^+^	0.152 (small)

a For all chi-square analysis ^a^ = 0.05. *^+^Significant at alpha, but significance lost when Bonferroni correction applied. *Significant at alpha, and significance retained when Bonferroni correct applied.

Finally, to understand how the use of certain study aids influenced 2023 students to draw a plausible product for the Unfamiliar Reaction, we conducted a logistic regression analysis. The students were prompted with a question on their online homework system (beSocratic) to select as many study aid choices they used to help them during their second semester of organic chemistry. The outcome of interest was binary, representing whether the OCLUE student selected the study aid (Yes’ or ‘No). Results are shown in Supporting Information S12 and S15.

## Results

### Research Question 1: How Do the Responses for the Familiar Reaction Vary over the Three Years (2018, 2022, and 2023)?

Just as in the Houchlei study,^18^ data for the familiar reaction were collected at two time points: 1) at the beginning of the spring semester, to capture what had been learned (or better put, retained) after the first semester of organic chemistry, and 2) at the end of the second semester, to investigate how a full year of organic chemistry impacts student arrow drawing and predictions.

### Results Time Point 1: Familiar Reaction

#### Finding 1a

After one semester of organic chemistry, OCLUE students from 2022 were significantly less likely than their peers from 2018, to predict a plausible product for the familiar acid catalyzed addition of water across an alkene.

**Figure 4:**
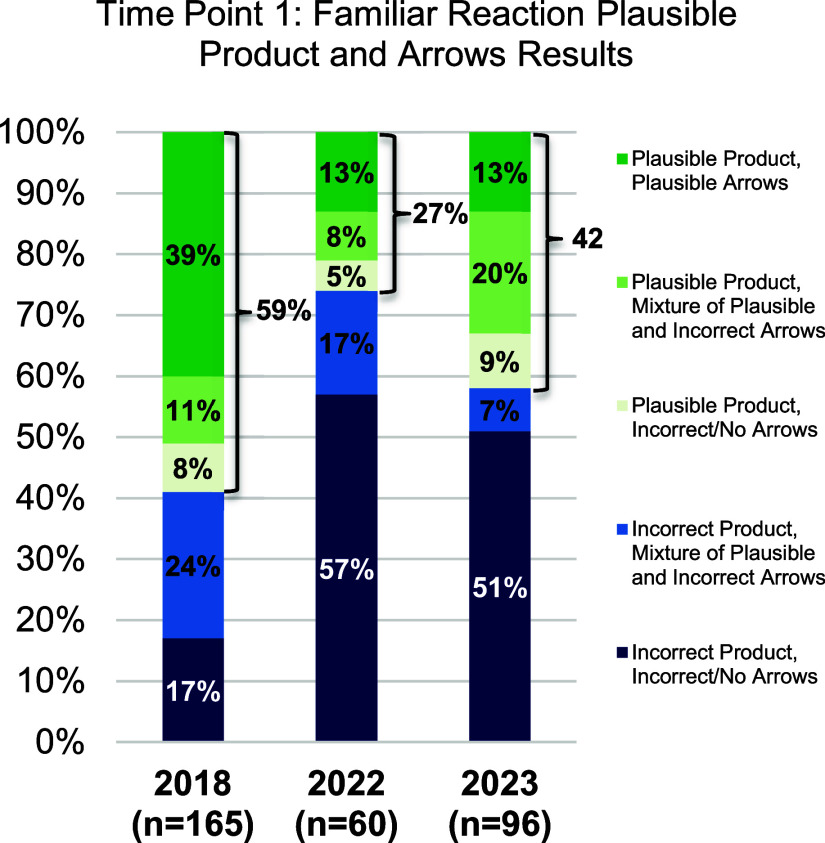
Percent of students who drew all mechanistic steps correctly, students who drew some mechanistic steps correctly, students who drew no mechanistic steps correctly, and students who got the incorrect product by year at time point 1 for the familiar reaction (beginning of the spring semester).

As shown in Figure 4, and Table 2 the only significant difference (after the Bonferroni correction) between the groups is for the percentage of plausible product between the 2022 students and the 2018 cohort, with a small to medium effect size. In general, it appears that after one semester students who learned organic chemistry during COVID appear to be at something of a disadvantage when compared with the in-person cohort from 2018.

#### Finding 1b

After one semester of organic chemistry, students from 2022 and 2023 cohorts are significantly less likely than their peers from 2018 to use mechanistic arrows to predict a plausible product for the familiar acid catalyzed addition of water across an alkene,

Table 3 compares the percent of students who drew a correct product with all plausible arrows (dark green in Figure 4). After the Bonferroni correction there are still significant differences between the in person OCLUE group (2018) and those from 2022 and 2023. That is, significantly fewer students use mechanistic arrows both during and post COVID to predict the product after one semester of organic chemistry.

**Table 3:** Time Point 1 Familiar Reaction: Comparison of Percent of Students Who Drew All Plausible Arrows and a Plausible Product For 2018, 2022, and 2023^a^

Cohort (count/total n)	Cohort (count/total n)	χ^2^ (df = 1)	p-value	Cramer’s V
2018, 39% (64/165)	2022, 13% (8/60)	14.176	<0.001*	0.251 (small – medium)
2018, 39% (64/165)	2023, 13% (12/96)	20.129	<0.001*	0.278 (small – medium)
2022, 13% (8/60)	2023, 13% (12/96)	0.001	0.970	

a For all chi-square analysis α = 0.05. *^+^Significant at alpha, but significance lost when Bonferroni correction applied. *Significant at alpha, and significance retained when Bonferroni correct applied.

### Results Time Point 2: Familiar Reaction

#### Finding 1c

After two semesters of organic chemistry, students who have taken all their chemistry courses online (2022 cohort) are significantly less likely to predict a plausible product for the acid catalyzed addition of water across an alkene, than their pre-COVID counterparts. However, students who participated in mostly in person or hybrid sections (2023 cohort) are just as likely to predict a plausible product. There is a caveat here that will be discussed later: in 2023 this activity was part of a final project that was also scored on good faith effort rather than correctness.

**Figure 5:**
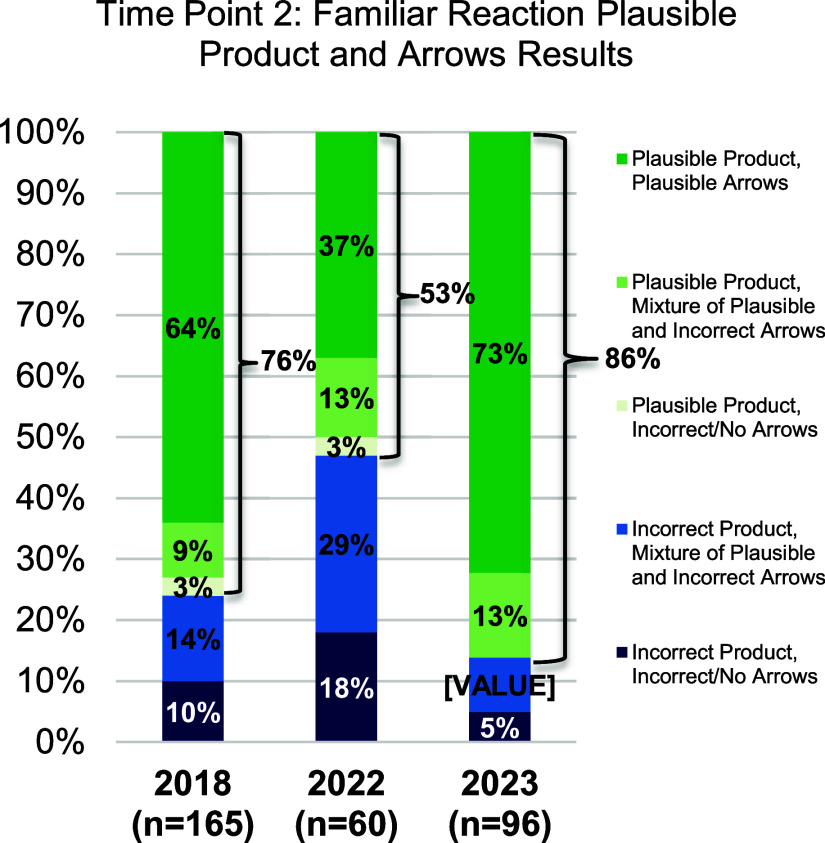
Percent of students who drew all mechanistic steps correctly, students who drew some mechanistic steps correctly, students who drew no mechanistic steps correctly, and students who got the incorrect product by year at time point 2 for the familiar reaction (end of the spring semester).

Figure 5 and Table 4 show the distribution of student responses and statistical analysis for Time Point 2. There is still a significant difference (with a small to medium effect size) between the online OCLUE cohort of 2022 and 2018. That is students who took all online chemistry courses were still less likely to correctly draw an appropriate product. There is also a significant difference (with a medium-large effect size) between 2022 and 23 after two semesters, whereas this was not the case after only one semester. The very large increase for 2023 may well be due to differences in the way that the activity was administered. While, in all cases students were told that they would receive credit for a good faith effort, in 2018 and 2022, the activity was administered as extra credit whereas in 2023 it was part of a final project (graded for good faith effort). We further discuss this finding in the Discussion.

**Table 4:** Time Point 2 Familiar Reaction: Comparison of Percent of Students Who Drew a Plausible Product for 2018, 2022, and 2023^a^

Cohort (count/total n)	Cohort (count/total n)	χ^2^ (df = 1)	p-value	Cramer’s V
2018, 76% (126/165)	2022, 53% (32/60)	11.160	<0.001*	0.223 (small – medium)
2018, 76% (126/165)	2023, 86% (83/96)	3.876	0.049*^+^	0.122 (small)
2022, 53% (32/60)	2023, 86% (83/96)	20.910	<0.001*	0.366 (medium – large)

a For all chi-square analysis α = 0.05. *^+^Significant at alpha, but significance lost when Bonferroni correction applied. *Significant at alpha, and significance retained when Bonferroni correct applied.

#### Finding 1d

After two semesters of organic chemistry, OCLUE students who have taken all their chemistry courses online (2022 cohort) are significantly less likely to use plausible arrows to predict a plausible product for the acid catalyzed addition of water across an alkene, than their pre-COVID counterparts. However, students who participated in mostly in person or hybrid sections (2023 cohort) are equally likely to use plausible arrows to predict a plausible product.

Table 5 shows the comparison of students who drew all plausible arrows on the way to their plausible product. In contrast to time point 1, where both 2022 and 2023 students were less likely to draw plausible arrows than the pre-COVID 2018 group, by end of two semesters (time point 2) the post COVID students (2023) have now caught up to their pre-COVID counterparts. However, the students who took all their chemistry courses during the pandemic still lag their pre- and post counterpoints.

**Table 5:** Time Point 2 Familiar Reaction: Comparison of Percent of Students Who Drew All Plausible Arrows and a Plausible Product for 2018, 2022, and 2023^a^

Cohort (count/total n)	Cohort (count/total n)	χ^2^ (df = 1)	p-value	Cramer’s V
2018, 64% (106/165)	2022, 37% (22/60)	13.642	<0.001*	0.246 (small – medium)
2018, 64% (106/165)	2023, 73% (70/96)	2.079	0.149	
2022, 37% (22/60)	2023, 73% (70/96)	20.054	<0.001*	0.359 (medium – large)

a For all chi-square analysis α = 0.05. *^+^Significant at alpha, but significance lost when Bonferroni correction applied. *Significant at alpha, and significance retained when Bonferroni correct applied

### Research Question 2: How Do the Responses for the Unfamiliar Reaction Vary over the Three Years (2018, 2022, and 2023)?

**Figure 6:**
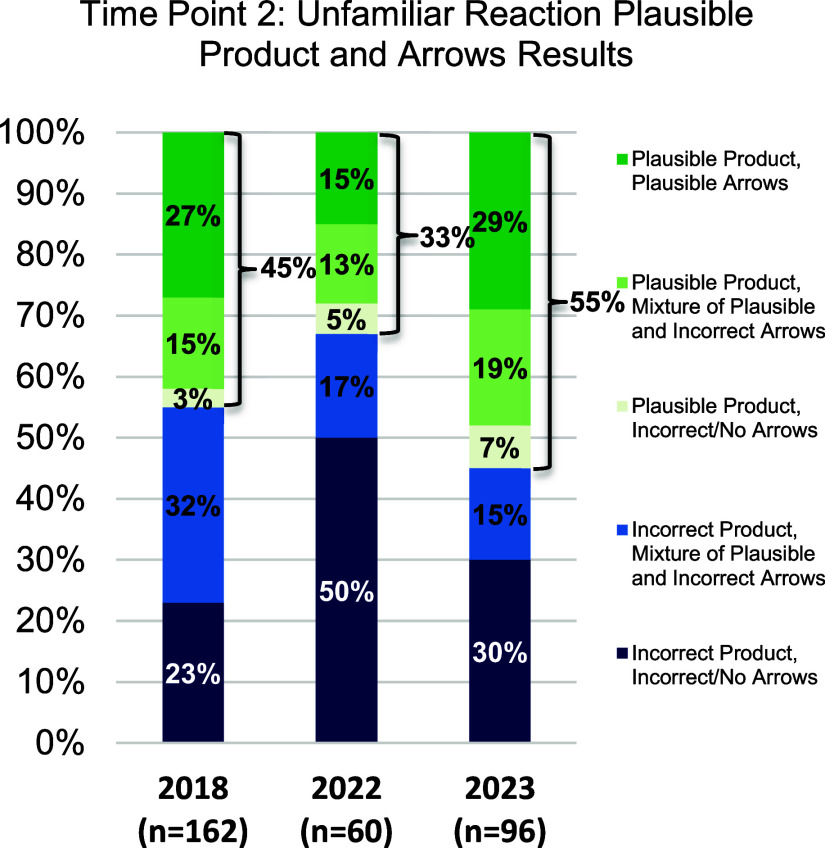
Percent of students who drew all mechanistic steps correctly, students who drew some mechanistic steps correctly, students who drew no mechanistic steps correctly, and students who got the incorrect product by year at time point 2 for the unfamiliar reaction (end of the spring semester).

As discussed earlier, the unfamiliar reaction involves a novel starting material that (to our knowledge) students had not seen before. This task requires students to use knowledge from reactions that they are more familiar with to predict how the unfamiliar starting material might behave. We have shown that students in traditional courses have a great deal of difficulty in proposing a plausible product for this reaction.^7,9^ Because of the difficulty of this task it is administered after two semesters of organic chemistry so that students have the requisite background knowledge and experience to determine the product. Figure 6 shows the percentage of students who predict a plausible product for the unfamiliar reaction (2018, 2022 and 2023).

#### Finding 2a

There is little difference between the percentage of students who draw a plausible unfamiliar product across all three years in this study.

As shown in Figure 6, and Table 6, the percentage of students who predict a plausible product for the unfamiliar reaction does not vary as widely as the familiar reaction. Although the percent of students from 2022 (online) is somewhat lower than for both 2018 and 2023 the small effect size significant difference for the 2022–2023 comparison disappears when the Bonferroni correction is applied.

**Table 6:** Time Point 2 Unfamiliar Reaction: Comparison of Percent of Students Who Drew a Plausible Product for 2018, 2022, and 2023^a^

Cohort (count/total n)	Cohort (count/total n)	χ2 (df = 1)	p-value	Cramer’s V
2018, 45% (73/162)	2022, 33% (20/60)	2.474	0.116	
2018, 45% (73/162)	2023, 55% (53/96)	2.484	0.115	
2022, 33% (20/60)	2023, 55% (53/96)	7.096	0.008*^+^	0.213 (small – medium)

a For all chi-square analysis α = 0.05. *Significant at alpha, but significance lost when Bonferroni correction applied. *^+^Significant at alpha, and significance retained when Bonferroni correct applied.

#### Finding 2b

There is little difference between the percentage of students who draw mechanistic arrows to predict an unfamiliar product across all three years in this study (Table 7).

**Table 7:** Time Point 2 Unfamiliar Reaction: Comparison of Percent of Students Who Drew All Plausible Arrows and a Plausible Product for 2018, 2022, and 2023^a^

Cohort (count/total n)	Cohort (count/total n)	χ^2^ (df = 1)	p-value	Cramer’s V
2018, 27% (44/162)	2022, 15% (9/60)	3.563	0.059*^+^	0.127 (small)
2018, 27% (44/162)	2023, 29% (28/96)	0.121	0.728	
2022, 15% (9/60)	2023, 29% (28/96)	4.096	0.043*^+^	0.162 (small)

a For all chi-square analysis α = 0.05. *Significant at alpha, but significance lost when Bonferroni correction applied. *^+^Significant at alpha, and significance retained when Bonferroni correct applied.

When we compare the percentage of students who drew all plausible arrows and a plausible product for the unfamiliar reaction, we again see little difference among the three groups. While there are small differences between 2022 and 2018, and 2022 and 2023, the significance is lost when the Bonferroni correction is applied

### Summary of Results

#### Research Question 1: How Do the Responses for the Familiar Reaction Vary over the Three Years (2018, 2022, and 2023)?

Using our two measures of analysis for the familiar reaction, we find that after one semester both students from 2022 and 2023 tend not to perform as well as those from 2018. However, by the end of two semesters of organic chemistry students from 2023 (post COVID) have caught up with their prepandemic counterparts, but the students from 2022 still performed at a lower level. Overall, there seems to be a decrease in performance during the COVID pandemic that is reversed after students return to in person classes.

#### Research Question 2: How Do the Responses for the Unfamiliar Reaction Vary over the Three Years (2018, 2022, and 2023)?

In contrast to the familiar reaction, there is no significant difference between students from all three cohorts for the unfamiliar reaction for the use of arrows to predict a plausible product.

## Discussion

In this study our goal was to determine the impact of the COVID pandemic and the subsequent return to in person instruction, on how students completed a set of organic mechanism tasks. These tasks have been the subject of several prior studies,^18,23−25^ thus our previously published data, provided us with the ability to compare cohorts across these time periods.

The two tasks we chose to study were (1) a task that was explicitly covered in each course: a familiar acid catalyzed addition of water to an alkene, and (2) a task that involved an unfamiliar (to our knowledge) starting material that would undergo reactions that had been discussed in each course. Our previously published data showed that almost all students were able to predict the product of the familiar reaction after two semesters of organic chemistry, and that students in the OCLUE transformed course were more likely than students in a traditional course to use mechanistic arrows to predict the product. However, in the case where the product cannot be memorized (since the starting material was novel to the students) the earlier study showed that students from the transformed course were more likely than traditional to use mechanistic arrows and more likely to predict a plausible product.^18,24^

In the present study, for reasons of accessibility to data and because traditional students have been shown in several studies to have great difficulty with unfamiliar tasks,^18,23^ we limited our comparison groups to students who were enrolled in the transformed OCLUE curriculum. For the familiar reaction, at time point 1 (after one semester), both the 2022 and 2023, performed significantly worse than their in-person peers from 2018. That is, they did not predict a plausible product as often, nor were they as likely to use mechanistic arrows. However, after two semesters, the two groups diverged. The students from 2022, who had experienced two years of online chemistry courses, were still significantly less likely to draw a plausible product, and to draw plausible mechanistic arrows, than the original in-person cohort of 2018, whereas by the end of two semester 2023 cohort showed a large increase in mechanism drawing and correct product.

There are several potential explanations for these differences. First, we consider the differences between 2022 and 2018. As discussed earlier, students in 2022 had taken all their chemistry courses online (all four semesters or general and organic chemistry), but had used the same text, learning objectives, homework assignments, and activities as those in 2018. They also had access to short recordings of content delivery, and class Zoom sessions were recorded and made available for those who could not attend in person. The activity studied here was given in the same format and was rewarded by the same amount of extra credit. Even so, it is clear that the performance of these students, whose two-year college chemistry experience had been completely online, had been impacted. The possible reasons for this are manifold: it does seem that some students learned less in the online format, but this could be because online learning is inherently more difficult, or maybe the online format led to a different class culture^24^ that was not as supportive as the in-person course, or that the stress and loneliness of the pandemic and its consequences had profound impacts on students. Indeed a 2022 survey of student health^31^ showed that students who reported tremendous, or much more than average stress, rose from around 50% before the pandemic to almost 70% in 2021. Additionally, changes in the grading criteria, and the move to credit/no credit may have impacted student motivation. Almost certainly it was a combination of these and other factors, and we are not alone in finding that student performance nosedived during the pandemic.^6^

However, if we compare the results from 2023 with 2022 for the familiar reaction, we see that the student performance returns to the prepandemic levels. For this academic year students had returned to full time class attendance, but, in spring 2023, because of campus violence,^28^ many students elected not to return to classes in person after the middle of February, and for the rest of that semester in person classes were also recorded and made available to students. Nevertheless, most students did attend classes in person for at least part of the semester and they had attended in person for the whole of the fall 2022 prior semester.

Interestingly, student performance at time point 2 in spring 2023 was better (although the difference is not significant) than any other data collection point that we have observed. We ascribe this to the fact that the activity was appended to the final project that replaced an in person final exam. Students were told that this final project was scored based on effort, just as the homework extra credits were, however it did count for a larger percentage (10%), whereas each homework extra credit counted for about 0.5% of the total grade. They had 4 weeks to complete the project that integrated three-dimensional tasks^32^ related to the semester content, in the context of a given amino acid (each student was assigned an amino acid). They were allowed to use any course materials and were asked to cite any external sources that they used. Despite the fact that this project was not graded for correctness, incorporating the activity on a required project almost certainly had an impact on some students’ performance. The familiar reaction is part of any organic chemistry course, and it would be reasonable to expect that if a student was uncertain about the mechanism and product, they would look it up in their course notes (although it should be noted that this is also true for homework assignments). However, as we discuss in the next section, this approach did not seem to help students with the unfamiliar reaction product and mechanism.

Despite the differences we observed for the known reaction, we did not see such marked differences for the unknown reaction. As shown in Findings 2a and b, the data for the students in the 2018, 2022, and 2023 cohorts are quite similar. Although there are small differences between 2022 and the 2018 and 2023 cohorts in this study, they are not significant after applying the Bonferroni correction. From this we conclude that for an unfamiliar task where students are required to use knowledge of mechanistic arrow drawing in a new situation, the percentage of students who are successful remains relatively constant, and that the modality, or circumstances of the course seems to have little impact on these particular students.

This brings us to the question of just who are these students who seem to be successful regardless of the circumstances in which the course is taken? We investigated a wide range of factors for students who drew a plausible product and those who did not, using a logistic regression analysis. While there is a small positive difference in course GPA, there was no other factor for which we could determine any significant effect, including the study techniques and materials student used, or personal identifying factors such as self-identified gender, or race. These analyses are provided in Supporting Information S12–S15.

## Implications for Teaching and Research

We believe there are several interesting and important findings that have emerged from the work that both confirm the widely reported learning losses during COVID but also give us cause for optimism during the post pandemic years. Our finding that students appear to be on a trajectory back to prepandemic performance is encouraging, especially since there are several reports of continued problems with student learning in math and STEM from the K-12 areas.^1,4,6^ However, we should not take this continued trajectory as a given, especially since students who spent their COVID years in the K-12 system and may have persistent learning gaps, are now finding their way onto to college campuses. It behooves us to continue to be vigilant and offer extra support when needed.

Additionally, the finding that more students appear to do better than ever for the known reaction on the final project for 2023 has potential ramifications for how we move forward to address the startling changes that will come as a response to the availability of Large Language Model Generative AI (GenAI) systems.^33^ In 2023, almost all the students produced an appropriate mechanism and plausible product for the acid catalyzed hydration of an alkene (the known reaction). We must presume that many of the students checked their work either online or in the course notes – and the advent of GenAI will make this easier and more seamless. Indeed, even before the advent of ChatGPT, a longitudinal study of student homework completion showed that the percent of students who did not benefit on the (secure) summative course assessments by doing homework rose from 14% in 2008 to over 50% in 2017. This lack of success appeared to correlate with the numbers of students who reported using Internet resources (copying and pasting answers) to do their homework.^34^ In our studies we have shown that far fewer students were able to predict and draw the mechanism of an unfamiliar product, even with the resources of the Internet available. At some point (soon) we are going to have to grapple with the idea that testing students on rather low-level tasks (such as giving the outcome of a familiar reaction) is almost certainly going to be circumvented by the ready access to AI systems.^35^ On the other hand, tasks that require students to use their knowledge in unfamiliar situations are much more difficult, and students who learn in a traditional format are unlikely to be able to handle such work.

Thus, one implication for research that emerges from this finding is that we need to work harder to determine what factors can support student success in unfamiliar tasks. While the transformed curriculum OCLUE, is more effective than the traditional curriculum in this endeavor, there are still around 50% of students who are unsuccessful on tasks that go beyond memorization or pattern recognition. As noted earlier, there is a great deal of effort devoted to improving outcomes in organic chemistry, but most of this effort has focused on pedagogies that increase student engagement such as using clickers, group work or flipped classrooms,^36,37^ which are also in use in OCLUE. However, since most of these studies are based on a traditional curriculum, it may be the grade increases seen in these studies might not apply to more complex tasks where students must use knowledge in an unfamiliar situation.

We suggest that it is time to move research beyond the difficulties students have in learning organic chemistry, on to the more productive path of exploring how to support student reasoning (and by implication mechanism drawing). One potentially useful approach is move beyond studies that focus on the EPF, and recognize that to use mechanistic arrows productively requires that students engage in mechanistic reasoning.^9,38,39^ That is, for students to draw mechanistic arrows successfully, they must understand that they are causal descriptions of how electrons are rearranging during a reaction or reaction step. For example, Crandell and Houchlei have shown that students who engage in writing causal mechanistic explanations about reaction mechanisms (SN1 and SN2) are also more likely to provide appropriate arrow pushing mechanisms for these same reactions.^25^

There have also been several studies that focus on supporting students to engage in reasoning about arrow drawing mechanisms, most of which rely on ways to scaffold such complex tasks in various ways.^15^ For example, Graulich and co-workers have published a number of papers where the investigate the effect of scaffolding mechanistic reasoning by using contrasting cases.^38,40^ That is, activities that require students to both recognize the similarities and differences between the cases and explain why they behave differently. Other approaches to scaffolding might include activities that emphasize the sequence of events that occur as a reaction proceeds, so that students are required to separate the mechanistic steps, thus lowering the cognitive load of the full sequence of a mechanism. Flynn and co-workers have designed a curriculum where students learn about mechanisms before they learn about functional groups, and has also emphasized that students must understand why these reactions occur in the way they do rather than simply memorizing them.^13^ We also need more studies that focus on helping students to use their knowledge in unfamiliar situations, and it may be that new curricula that focus on nontraditional aspects of organic chemistry such as bio-organic chemistry,^41^ systems thinking and green chemistry^42^ could be particularly important in this endeavor. Ideally, we would like all students who complete our courses to be able to use their knowledge in unfamiliar situations, after all we might say that is the purpose of education. We have clearly made steps along this path, but there is still work to be done.

## Limitations

There are several limitations to this study. Our choice of the two reactions (familiar and unfamiliar) is determined by the fact that we had a large corpus of earlier data on these two cases. Clearly if we had collected data across a wider range of reactions our conclusions might be different. Another limitation of this study is that it was conducted in a single university. Although we attempted to control some variables (the curriculum, the learning goals and learning materials, and the instructor all being the same for each iteration) the ongoing changes in modality and campus situations means we are comparing three different cases in which there are more differences than just those associated with a move to online instruction during COVID. Another factor that could impact findings is the different number of students in each cohort: to ameliorate this we have used a very conservative approach to identifying differences and the numbers in each cohort are still large enough for statistical comparisons.

Ideally, a study such as this would be designed in such a way that all the potential variables are controlled. In our study this was not possible (scheduling problems, campus violence, changes in how the activity was perceived by students (extra credit vs part of a larger activity graded for effort but not correctness). However, natural experiments^43^ done “in the real world” as opposed to a cognitive laboratory situation have value, since this is where we teach and learn.

## Supporting Information Available

Study participant demographics, reaction coding, and inter-rater reliability (PDF, DOCX)
