# Supplementary material for: Investigation into the Impact of Online Learning and the Pandemic on Student Use of Mechanistic Arrows
Source: J Chem Educ. 2025 Apr 8;102(5):1755–64. doi: 10.1021/acs.jchemed.4c01274 (PMC12080246; doi:10.1021/acs.jchemed.4c01274)
Supplement: Supplementary file 1 — ed4c01274_si_001.pdf [file ed4c01274_si_001.pdf]

An Investigation into the Impact of Online Learning and the Pandemic on Student Use of Mechanistic Arrows

Veeda Scammahorn[†], Samantha Houchlei[‡], Hunter Williams, Melanie M Cooper* [†]

[†] Department of Chemistry, Michigan State University, 578 South Shaw Lane, East Lansing, Michigan 48824, United States

[‡] Department of Chemistry, University of Minnesota Twin Cities, Minneapolis, Minnesota 55455, United States.

Supporting Information

STUDIES PARTICIPANT DEMOGRAPHICS, REACTION PROMPTS, AND INTERRATER RELIABILITY

STUDENT DEMOGRAPHICS

S1. Mann-Whitney Comparison of OCLUE student cohorts (2018, 2022, and 2023) for ACT Math Scores

Cohort (n=total)	Mean	Mann-Whitney U	Z	p-value	Effect Size r
Online OCLUE 2022 (n=59)	27.19	4834.000	-0.608	0.543	
In-Person OCLUE 2018 (n=173)	27.01				
Hybrid OCLUE 2023 (n=68)	26.25	5200.000	-1.405	0.160	
In-Person OCLUE 2018 (n=173)	27.01				
Online OCLUE 2022 (n=59)	27.19	1693.000	-1.520	0.129	
Hybrid OCLUE 2023 (n=68)	26.25				

S2. Mann-Whitney Comparison of OCLUE student cohorts (2018, 2022, and 2023) for Prior GPA Scores

Cohort (n=total)	Mean	Mann-Whitney U	Z	p-value	Effect Size r
Online OCLUE 2022 (n=60)	3.52	2992.500	-4.256	<0.001	0.279
In-Person OCLUE 2018 (n=173)	3.60				
Hybrid OCLUE 2023 (n=94)	3.67	7391.000	-1.231	0.218	
In-Person OCLUE 2018 (n=173)	3.60				
Online OCLUE 2022 (n=60)	3.52	2278.500	-2.014	0.044	0.162
Hybrid OCLUE 2023 (n=94)	3.67				

S3. Description of Binary Gender of OCLUE student cohorts (2018, 2022, and 2023)

Cohort (n=total)	Male (n)	Female (n)	χ2 (df=1)	p-value	Cramer's V
Online OCLUE 2022 (n=60)	13% (8)	87% (52)	6.142	0.013	0.162
In-Person OCLUE 2018 (n=173)	29% (51)	71% (122)			
Hybrid OCLUE 2023 (n=94)	35% (33)	65% (61)	0.894	0.344	
In-Person OCLUE 2018 (n=173)	29% (51)	71% (122)			
Online OCLUE 2022 (n=60)	13% (8)	87% (52)	8.887	0.003	0.240
Hybrid OCLUE 2023 (n=94)	35% (33)	65% (61)			

S4. Description of Self-Identified Ethnicity of OCLUE student cohorts (2018, 2022, and 2023)

Cohort (n=total)	White (n)	Non-White (n)	χ2 (df=1)	p-value	Cramer's V
Online OCLUE 2022 (n=60)	67% (40)	33% (20)	2.139	0.144	
In-Person OCLUE 2018 (n=173)	76% (132)	24% (41)			
Hybrid OCLUE 2023 (n=94)	71% (67)	29% (27)	0.810	0.368	
In-Person OCLUE 2018 (n=173)	76% (132)	24% (41)			
Online OCLUE 2022 (n=60)	67% (40)	33% (20)	0.367	0.545	
Hybrid OCLUE 2023 (n=94)	71% (67)	29% (27)			

S5. Description of Intended Major for OCLUE student cohorts (2018, 2022, and 2023)

Cohort (n=total)	Preprofessional and Health Sciences	Animal and Plant Sciences	Physical Science and Engineering	Other
In-Person OCLUE 2018 (n=173)	74%	18%	2%	6%
Online OCLUE 2022 (n=60)	88%	10%	0%	2%
Hybrid OCLUE 2023 (n=94)	74%	24%	1%	1%

REACTION PROMPTS

S6. Familiar Reaction Prompt

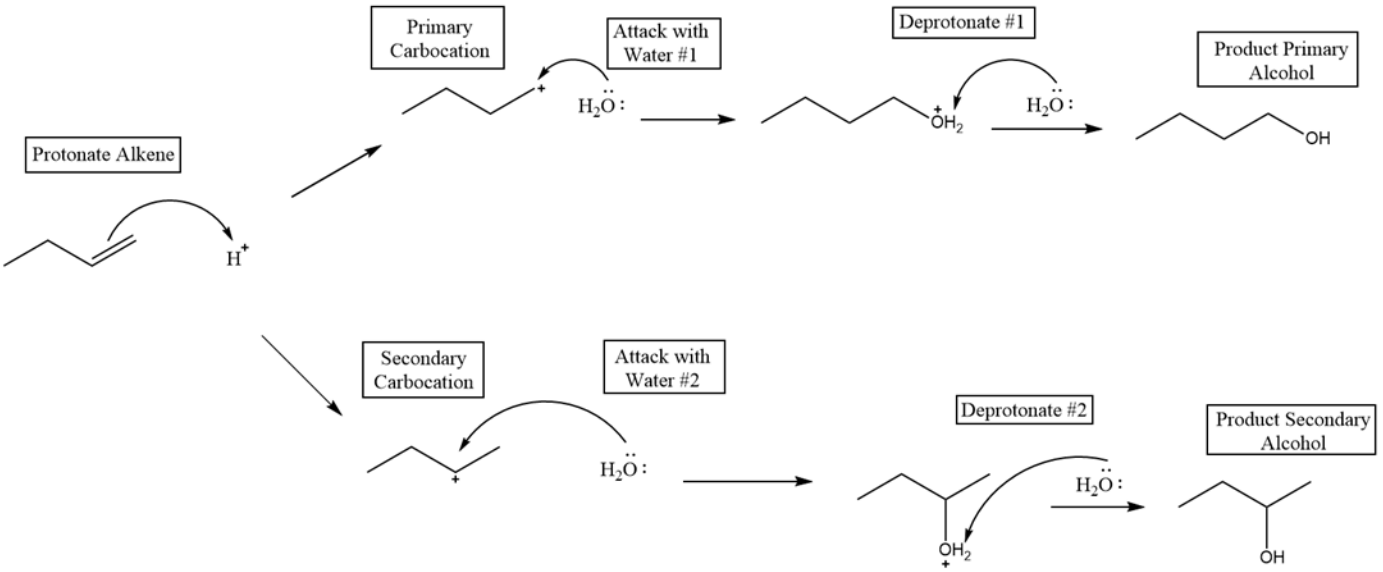

S7. Unfamiliar Reaction Prompt

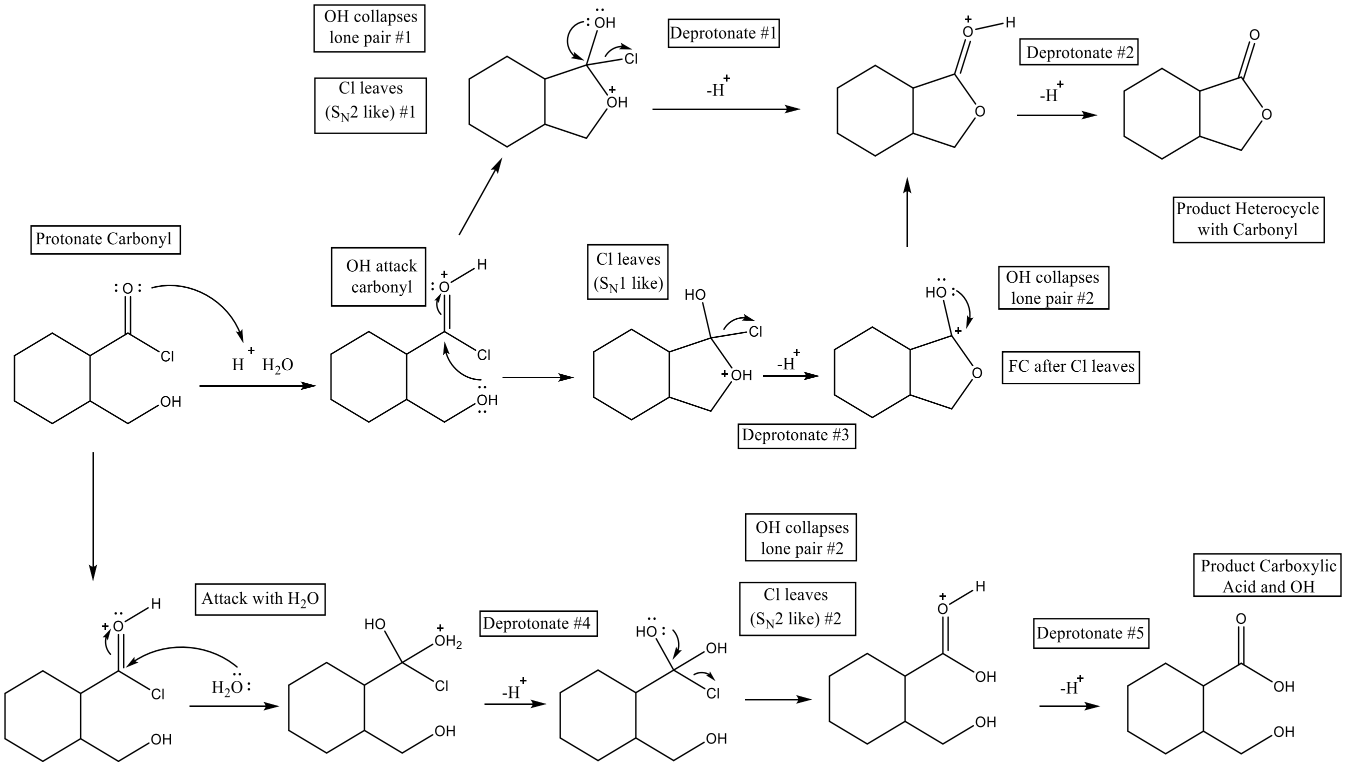

INTERRATER RELIABILITY

S8. Familiar Reaction for OCLUE 2022 Students

Code (n=60)	Kappa
Protonate Alkene	0.90
Primary Carbocation	1.00
Secondary Carbocation	0.93
Attack with Water #1	0.96
Attack with Water #2	0.87
Deprotonate #1	1.00
Deprotonate #2	0.83
Product Primary Alcohol	0.96
Product Secondary Alcohol	0.80
No Mechanism Only Product	0.82
Drew Product First Then Mechanism	0.96

S9. Familiar Reaction for OCLUE 2023 Students

Code (n=60)	Kappa
Protonate Alkene	0.86
Primary Carbocation	0.97
Secondary Carbocation	0.90
Attack with Water #1	0.93
Attack with Water #2	1.00
Deprotonate #1	0.97
Deprotonate #2	0.82
Product Primary Alcohol	1.00
Product Secondary Alcohol	0.86
No Mechanism Only Product	0.92
Drew Product First Then Mechanism	1.00

S10. Unfamiliar Reaction for OCLUE 2022 Students

Code (n=60)	Kappa
Protonate Carbonyl	0.83
OH Attack Carbonyl	0.78
H2O Attack	0.90
OH Collapses Lone Pair #1	0.92
Cl leaves (Sn2 like) #1	0.97
Cl leaves (Sn1 like)	0.91
OH collapses lone pair #2	1.00
FC after Cl leaves	1.00
Deprotonate #1	0.95
Deprotonate #2	0.92
Deprotonate #3	0.87
Deprotonate #4	0.93
Deprotonate #5	0.89
Heterocycle with Carbonyl Product	0.82
Carboxylic Acid and OH Product	0.87
No Mechanism Only Product	0.96
Drew Product First Then Mechanism	1.00
Protonate Carbonyl	0.94
OH Attack Carbonyl	0.91

S11. Unfamiliar Reaction for OCLUE 2023 Students

Code (n=60)	Kappa
Protonate Carbonyl	0.88
OH Attack Carbonyl	0.82
H2O Attack	0.93
OH Collapses Lone Pair #1	0.89
Cl leaves (Sn2 like) #1	0.96
Cl leaves (Sn1 like)	0.95
OH collapses lone pair #2	0.97
FC after Cl leaves	0.98
Deprotonate #1	0.94
Deprotonate #2	0.88
Deprotonate #3	0.92
Deprotonate #4	0.95
Deprotonate #5	0.92
Heterocycle with Carbonyl Product	0.86
Carboxylic Acid and OH Product	0.94
No Mechanism Only Product	0.96
Drew Product First Then Mechanism	0.92
Protonate Carbonyl	0.98
OH Attack Carbonyl	0.95

LOGISTIC REGRESSIONS FOR OCLUE 2022 AND OCLUE 2023 STUDENTS

S12. OCLUE 2022 Students Logistic Regression Results for Drawing a Plausible Product for the Unfamiliar Reaction

	B	S.E.	Wald	df	Sig.	Exp(B)
Ethnicity	-0.108	0.643	0.028	1	0.866	0.897
Gender	-1.623	0.856	3.600	1	0.058	0.197
Math Scores	0.080	0.095	0.720	1	0.396	1.083
Prior GPA	2.242	1.815	1.526	1	0.217	9.411
Constant	-10.032	6.427	2.437	1	0.119	0.000

S13. OCLUE 2023 Students Logistic Regression Results for Drawing a Plausible Product for the Unfamiliar Reaction

	B	S.E.	Wald	df	Sig.	Exp(B)
Ethnicity	0.619	0.479	1.669	1	0.196	1.857
Gender	-0.958	0.470	4.158	1	0.041	0.384
Math Scores	0.105	0.058	3.267	1	0.071	1.110
Prior GPA	1.067	0.656	2.645	1	0.104	2.906
Constant	0.456	0.474	0.926	1	0.336	1.577

S14. OCLUE 2023 Students Logistic Regression Results for Drawing a Plausible Product and Selection of Study Aids for the Unfamiliar Reaction

Students have used study aids and will continue to do so in future courses. To understand how the use of certain study aids influenced our OCLUE 23 students to draw a plausible product for the Unfamiliar Reaction, we conducted a logistic regression analysis. The students were prompted with a question on their online homework system (beSocratic) to select as many study aid choices they used to help them during their second semester of organic chemistry. The outcome of interest was binary, representing whether the OCLUE student selected the study aid (Yes' or 'No). Logistic regression was chosen as the appropriate analysis method because it models the log odds of the outcome, providing a more accurate and interpretable approach for binary outcomes. The independent variables are the Study Aids which includes some examples such as 'Attended 'Lectures', 'TA Office Hours', 'Extra Problems Sets on Web' and more. Logistic regression also allowed us to estimate odds ratios, providing a clear interpretation of the strength and direction of the relationships between the predictors and the likelihood of a student drawing a plausible product for the unfamiliar reaction. If a significant value was found in the Logistic Regression Analysis, a contingency table is provided to showcase frequency distribution of the OCLUE 23 Students who drew a plausible product for the unfamiliar reaction and the significant independent variable.

S15. Contingency Table Showing the Relationships Between 'Reviewed Recitation Keys' and Students Who Drew a Plausible Product for the Unfamiliar Reaction

	B	S.E.	Wald	df	Sig.	Exp(B)
Attended Lectures	0.516	0.579	0.794	1	0.373	1.675
Pre-Class Lecture Videos	-1.455	1.824	0.637	1	0.425	0.233
Class Recordings	0.235	0.572	0.168	1	0.682	1.264
Course Textbook	0.553	0.610	0.820	1	0.365	1.738
TA Office Hours	0.263	0.759	0.120	1	0.729	1.300
Materials on Other Websites	0.210	0.484	0.188	1	0.665	1.233
Private Tutor	-0.94	1.208	0.006	1	0.938	0.910
Extra Problem Sets on Web	1.695	1.345	1.587	1	0.208	5.447
Materials from Other Courses	-0.348	0.669	0.271	1	0.603	0.706
Reviewed Mock Exam Keys	-21.444	40192.919	0.000	1	1.000	0.000
Reviewed Recitation Keys	-1.656	0.566	8.567	1	0.003	0.191
QandA Forums	-2.713	1.615	2.822	1	0.093	0.066
Other Textbooks	22.912	23720.178	0.000	1	0.999	8920727164.8
Actively Took Lecture Notes	-0.811	0.738	1.208	1	0.272	0.444
Constant	24.165	40192.919	0.000	1	1.000	31251609157

	Plausible Product (1)	Incorrect Product (0)	Total
Yes Recitation (1)	31	37	68
No Recitation (0)	22	6	28
Total	53	43	96

REFERENCES

1. IBM Corp. SPSS Statistics for Windows; IBM Crop.: Armonk, NY, USA, 2017.
2. Green, S.; Salkind, N. Using SPSS for Windows and Macintosh: Analyzing and Understanding Data; Pearson Education Inc: Boston, MA, USA, 2010.

3. Cohen, J. A Coefficient of Agreement for Nominal Scales. Educ. Psychol. Meas. 1960, 20 (1), 37–46. https://doi.org/10.1177/001316446002000104.
4. Grove, N. P.; Cooper, M. M.; Rush, K. M. Decorating with Arrows: Toward the Development of 115 Representational Competence in Organic Chemistry. J. Chem. Educ. 2012, 89 (7), 844–849. https://doi.org/10.1021/ed2003934.
5. Houchlei, S. K.; Bloch, R. R.; Cooper, M. M.; Rush, K. M. Mechanisms, Models, and Explanations: Analyzing the Mechanistic Paths Students Take to Reach a Product for Familiar and Unfamiliar Organic Reactions. J. Chem. Educ. 2021, 98 (9), 2751-2764. https://doi.org/10.1021/acs.jchemed.1c00099.
